# Composition Classification of Ultra-High Energy Cosmic Rays

**DOI:** 10.3390/e22090998

**Published:** 2020-09-07

**Authors:** Luis Javier Herrera, Carlos José Todero Peixoto, Oresti Baños, Juan Miguel Carceller, Francisco Carrillo, Alberto Guillén

**Affiliations:** 1 Computer Architecture and Technology Department, University of Granada, 18071 Granada, Spain; oresti@ugr.es (O.B.); franciscocp@ugr.es (F.C.); aguillen@ugr.es (A.G.); 2 Department of Basic Science and Environment, University of São Paulo, Lorena - SP 12602-810, Brazil; toderocj@usp.br; 3 Theoretical and Cosmos Physics Department, University of Granada, 18071 Granada, Spain; jmcarcell@ugr.es

**Keywords:** cosmic rays, ultra high energy, mass composition, feature selection, deep learning

## Abstract

The study of cosmic rays remains as one of the most challenging research fields in Physics. From the many questions still open in this area, knowledge of the type of primary for each event remains as one of the most important issues. All of the cosmic rays observatories have been trying to solve this question for at least six decades, but have not yet succeeded. The main obstacle is the impossibility of directly detecting high energy primary events, being necessary to use Monte Carlo models and simulations to characterize generated particles cascades. This work presents the results attained using a simulated dataset that was provided by the Monte Carlo code CORSIKA, which is a simulator of high energy particles interactions with the atmosphere, resulting in a cascade of secondary particles extending for a few kilometers (in diameter) at ground level. Using this simulated data, a set of machine learning classifiers have been designed and trained, and their computational cost and effectiveness compared, when classifying the type of primary under ideal measuring conditions. Additionally, a feature selection algorithm has allowed for identifying the relevance of the considered features. The results confirm the importance of the electromagnetic-muonic component separation from signal data measured for the problem. The obtained results are quite encouraging and open new work lines for future more restrictive simulations.

## 1. Introduction

Particles with cosmic origin that reach the Earth are known as Cosmic Rays (CR). There are many unknowns in their study, although there are two important major aspects that, once resolved, could provide useful information for astrophysicists. The first one is their origin, when considering aspects, such as how they were produced, accelerated, and their propagation through the galactic and extra-galactic medium. The second aspect that make their study so important is that the energy density of cosmic radiation is of the same order of magnitude as the one found in magnetic fields and stars, so they could give a hint on the total energy balance of our universe. There is a third aspect, which is known as the Greisen—Zatsepin—Kuzmin (GZK) limit [1], which is an abrupt drop in cosmic ray flux to energies above $10^{19.5}$ eV, and, from this, it seems that the universe is “opaque” for events with energies above this limit.

For energies above the solar modulation spectrum (10 GeV/nucleon) [2], the cosmic rays are called “high energy” ones and, for energies above $10^{16}$ eV, they are called “ultra high energy” (UHE) cosmic rays. Three quantities can be used to describe a cosmic ray caused by an incident particle (called primary) in the earth’s atmosphere: the energy, the angle of arrival (θ), and the type (mass and charge). All of the observatories of cosmic radiation can measure the energy and θ for each event, but, up to now, there is no way to measure the mass directly. Using extrapolated data from particle accelerators, the particle astrophysics community developed models and simulators that only allowed for us to propose probabilities of mass compositions, in distributions well-defined for bins in energy and θ [3]. The classification of the particle composition is a crucial question that should be answered in order to better understand the three aspects described above.

Beyond that, knowing the composition information of each event will make it possible to search for flux of protons at the highest energies [4]. Therefore, it could improve previous particle-physics studies at 10 EeV and extend them to energies as high as 200 TeV (center of mass). Extending composition sensitivity to all possible energy ranges and a larger range of zenith angles will provide almost an order of magnitude increase in statistics to resolve the question of the origin of the flux suppression (GZK limit).

The observation of ultra high energy cosmic rays (UHECR) is based on the detection of secondary particles cascades that are produced in the atmosphere, resulting from the initial collision of the primary with some molecule of the air (usually $N_{2}$) at the top of the atmosphere (around 35 km altitude). Around $10^{10}$, secondary particles arrive at ground level distributed within a radius of up to 3.000 meters. This phenomenon is known as extensive air shower (EAS), and it was first measured by Pierre Auger in 1939 [5]. The detectors are not able to discriminate the secondary particles; they measure a signal that relates the cascade’s energy deposit and its evolution over time. Analysis of this signal is the source of the cascade and the primary particle information.

This paper tackles the problem of using Machine Learning (ML) to identify the type of particle that generates the cascade due to the importance of this information, which can be extracted from a EAS. A recent work has performed a first approach to the composition identification problem by estimating the muonic number in simulated traces [6]. Monte Carlo models predict that heavier primaries (such as Nitrogen or Iron) have more muons than lighter primaries (such as protons or Helium). However, even knowing this element accurately, the mapping of this feature to a particle type still remained unsolved.

A preliminary work [7], first dealt with this question by trying to tackle the problem using two simple deep learning models, approaching the problem both as a classical classification and as a continuous regression-like output. However due to the limitations of the dataset, limits in the classification accuracy attainable by ML models were not verified and a wider and more thorough study was needed. Thus, the aim of this work is to go in depth into the limits in the possibility of identifying the type of particle that generated an EAS, from a set of ideal measurements at ground level. Moreover, the importance of the different factors that are involved in a EAS for the primary identification will be assessed by an effective feature selection specific technique.

For that, this work uses simulated ground truth for several features (like the muon and electromagnetic numbers) using a data set generated with CORSIKA (**C**Osmic **R**ay **SI**mulations for **KA**scade) simulator [8,9]. Five different types of particles have been considered: Photons, Protons, Helium, Nitrogen, and Iron. Four different machine learning classifiers have been trained and analyzed under Python implementation, including XGBoost, K-NN, Deep Neural Networks and Support Vector Machines. This comparison allows comparing these alternatives, both from the performance and the computational cost point of view, allowing for us to assess the best alternative for the given problem. Moreover, a modification of the Markov Blanket Mutual Information Feature Selection (MBFS) algorithm [10,11,12] adapted for classification has been applied in order to identify the relevance of the features involved. The importance of this type of ML techniques application comparative analysis is corroborated in the extent recent literature for other problems from a wide range of fields [13,14,15,16].

The rest of the paper is organized, as follows: Section 2 presents the data used in the experiments. Section 3 introduces the classifiers and feature selection algorithm proposed for this work. Section 4 presents the experiments and show the results that were obtained for the problem. Section 5 discusses the results. Finally, conclusions are drawn in Section 6.

## 2. Data Description

The data used in this research were generated by the CORSIKA Monte Carlo code, which is a particle interaction simulator designed to extrapolate hadronic interactions (hadrons are particles with internal structure, such as protons, helium, carbon, etc.) with center of mass energies above 100 TeV. To get an idea of the importance of this simulator, the LHC-CERN collider has a maximum energy of 6.5 TeV per beam (by the end of 2018) [17] and this is the limit (until now) of the experiments in particle physics. There is no actual data describing interactions above 100 TeV, which is the typical collision energy of cosmic particles with our atmosphere. This is where the need for a simulator with extrapolations of hadronic interaction models comes from.

The simulations are done by tracking the particles through the atmosphere until they undergo reactions with the air nuclei and produce a cascade of the secondary particles. These cascades can be described in a simplified way as the composition of three components: a hadronic cascade (heavier particles, such as pions, neutrons, and protons), a muonic cascade (muons are produced by the pions decay, and their mass is about 200 times greater than the electron mass), and an electromagnetic cascade (photons, electrons, and positrons). The output of the program is a dataset with the information of all particles of the cascade. Each particle is assigned with seven information: position (x, y, z), energy (px, py, pz), and type.

The Monte Carlo code divide the development of the cascade in three types of interaction models to describe the cascade particles: high energy (above 100TeV), low energy (below 100 TeV), and electromagnetic interactions. The code chooses one of these three models based on the energy and type of the particle over the course of development.

The code also provides several options for types of interaction models. For high energy, the models are based on the calculation of the cross section of the secondary particle scattering, the hadron mini-jets. Each model considers a different treatment for the partons (fundamental particles that constitute a hadron) and a distinct phase space. All of the models use the quantum field theory of Gribov–Regge, which is a model used to describe the interaction between hadrons. The models QGSJetII-04 (**Q**uark **G**luon **S**tring model with **Jet**s) [18], SIBYLL [19], and EPOS(LHC) (**E**nergy conserving quantum mechanical multi-scattering approach, based on **P**artons, **O**ff-shell remnants and **S**plitting parton ladders) [20] are options that can be used to describe high energy collisions. At lower energies, interactions can be used the models GHEISHA (**G**amma **H**adron **E**lectron **I**nteraction **SH**ower) [21], the FLUKA [22], or the microscopic URQMD (**U**ltra-**R**elativistic **Q**uantum **M**olecular **D**ynamics) [23]. For electromagnetic (EM) interactions, a version of the code EGS4 (**E**lectron **G**amma **S**hower) [24] or the analytical NKG (**N**ishimura-**K**amata-**G**reisen) [2] formulas may be used. For this work. we are using, at higher energy, the model QGSJetII-04, combined with FLUKA2011.2c for lower energies, and EGS4 for EM interactions.

We simulate a set of events ($1.2\times 10^{4}$) for each primary particle mass (photon (no mass), proton, helium, nitrogen, and iron—total: $6\times 10^{4}$ events) and within this set we randomize, for each event, the values of energy ($10^{18.5}$ up to $10^{19.0}$ eV), angle of entry into the atmosphere (θ: 0 until 60 degrees), and the mean free path for first collision ($X_{0}$). The errors are related with the systematic of this randomization, which was performed using a Monte Carlo procedure.

Some of the factors that can be extracted from the output dataset for each simulation like $X_{max}$ (the atmospheric depth (g/cm${}^{2}$) where the cascade have the maximum number of particles) and ZFirstm (altitude where the particle starts to interact with the atmosphere [m]) are difficult to be measured. There are few real data measurements for $X_{max}$ and, to date, it is not possible to measure it at ground level, especially for events at high energies. Therefore, $X_{max}$ and ZFirstm were discarded in order to provide a more realistic, still optimistic, definition of the type of data that can be measured at ground level. Subsequently, the features considered for the work were:

- NALLParticlesTotal: total number of particles generated by the event at the ground level.
- MUTotal: total number of muons, at the ground level.
- ELTotal: total number of electromagnetic particles, at the ground level.
- Zenith: zenith angle of the primary particle [degrees].
- Energy: primary particle energy [GeV].

Being precise on the information provided by the CORSIKA simulator, there is no chance to know, with accuracy, the total number of particles reaching the Earth surface. However, an estimation of it can be provided that could be accurate enough to use those values for the classification [7]. The same can be said for the Energy, and muonic and electromagnetic signals. Dataset and code used for the work presented in this paper can be downloaded from (https://github.com/aguillenATC/Entropy-CompositionClassificationUHECR).

## 3. Methods

This section details the workflow followed in order to tackle the problem. All of the compared classification methodologies are briefly discussed; similarly, the feature selection phase, in which the importance of the features studied is analyzed, is also described. The methodology follows the same steps than the ones proposed in [25] for hurricane intensity estimation.

### 3.1. Classification Methods

Four different classifiers have been trained and tested in order to verify the possible effectiveness of an intelligent method applied to the prediction of the type primary reaching the atmosphere from a set of ideal measurements. These four methods are: Artificial Neural Network, Gradient Boosting—XGBoost, Support Vector Machines, and K-nearest neighbors. These techniques and the training process for each of them, are briefly described next. Finally, the computational framework carried out for a fair comparison the four methodologies is presented.

#### 3.1.1. Artificial Neural Network

Deep Neural Network (DNN) with a Feedforward architecture was utilized. This type of model is nowadays the state of the art in many complex problems, specially in those with stochastic nature [26]. The capability of these type of models to deal with large data sets has been improved, thanks to stochastic optimization algorithms.

Regarding the architecture and topology of the network, a number of possibilities for number of layers and of unit per layer were assessed, in a random manner, in order to finally select the optimal network configuration. The selected learning and network architecture parameters were:

1. Number of layers: configurations containing from 2 up to 7 hidden layers were considered. ReLu units were taken for the these [27]. For the output layer, softmax units (one per class) were used.
2. Number of neurons: configurations containing from five up to 50 neurons per layer were considered for the hidden layers.
3. Constant weight initialization to 0.025 (for the sake of reproducibility).
4. Optimisation algorithm: Adam [28] with default parameters (after analysing the behaviour of higher and lower learning rate and beta values) and a maximum of 500 epochs. Batch size was set fixed to 256.
5. Loss function: crossentropy for classification [29].

#### 3.1.2. XGBoost

XGBoost (eXtreme Gradient Boosting) [30] is an efficient algorithm implementing a regularized version of Gradient Boosting algorithm. In summary, this type of learning algorithm, iteratively optimize a set of tree models, each learning the residual from the previously optimized set of models, in a gradient descent manner. XGBoost is a widely used implementation of gradient boosting, which has reached great rankings in many machine learning competitions, for instance, those proposed at Kaggle (Kaggle- Data Science Projects. https://www.kaggle.com/competitions).

Python implementation of XGBoost (v.0.72) (XGBoost- Python library. https://xgboost.readthedocs.io/en/latest/python/index.html.) was used for the simulations. Hyperparameters optimized included were maxdepth (varying from two to five) and eta (varying from 0.05 to 1 in steps of 0.05); a fixed number of rounds for the training process was set to 150.

#### 3.1.3. Support Vector Machines

SVMs have, for many years, been the the most frequently used classification paradigm in machine learning until the emergence of Deep Learning few years ago [31]. Binary SVM classification deals with the identification of the optimal largest margin classification hyper-plane in a dual space in order to separate the two classes involved. Multiclass classification in SVM is normally performed through the construction of k(k−1)/2 classifiers, being k the number of classes, each one training a separating hyper-plane for two different classes. Subsequently, a voting scheme is used to identify the class to which each pattern belongs. Among the kernel functions alternatives, Gaussian Radial Basis Function kernel has been chosen, as it has proven to offer a good asymptotic behavior [32]. The estimation of the hyper-parameters (σ and γ) of the SVM was done using grid search and cross-validation.

#### 3.1.4. *K*-Nearest Neighbors

*K*-Nearest Neighbors (KNN) is a simple and fast classification algorithm, nevertheless attaining comparable results to other more complex machine learning techniques in many real problems. It is based on the search of the (*k*) most similar samples of a new sample, and assigning the most frequent class among the neighbors to it. There is a number of distance or similarity measures for the identification of the neighborhood of a sample, although the Euclidean distance is the most used one to deal with continuous features. Grid-search was used over the training dataset in order to find an optimal value for k∈[0,50].

#### 3.1.5. Classifiers Comparison

The four methodologies were compared both in terms of accuracy and training computational complexity. Different configurations of the hyperparameters for each algorithm were assessed over a training dataset made up by 80% of the data, using a five-fold cross-validation scheme over the training dataset for optimization. Subsequently, given an optimal hyperparameter selection, a later five-fold cross-validation assessment process over the whole dataset was used for performance comparison of the different classification alternatives. i.e. training using optimal hyperparameters selected on four folds, and testing over the remaining one, in a cross-validation way, finally providing mean and std accuracies.

A similar number of evaluations of the respective hyperparameters alternatives was observed in order to provide a fair comparison over the different hyperparameters optimization for each classification paradigm. Thus, for SVM, nine different values (using logarithm scaling) for each σ and γ were assessed (81 configurations); for XGBoost, four different values for maxdepth and 20 possible values for eta were assessed (80 configurations); for DNN, 80 random network architectures (in terms of number of layers and number of hidden units per layer) under the specified parametrization were assessed; finally, for KNN classification, 50 different values of *k* were tested (we considered that assessing larger values of k was pointless). The computational cost of the training procedure and training and test accuracy were compared for the four alternatives.

### 3.2. Markov Blanket Feature Selection (MBFS) Algorithm

Feature selection is a key preprocessing step in any classification problem [33]. Although, in this problem, a reduced number of features is present, it is still very important to identify their relevance order and consequent performance. Among the wide literature on feature selection, we have chosen the mutual information (MI) criterion from Information Theory [34], due to its capability of identifying nonlinear relationships between the features involved.

The estimation of MI among features is a key element for the performance of feature selection algorithms based on this criterion. This work uses the k-nearest neighbor estimator that was proposed by Kraskov et al. in [35], as the method proved to behave robustly, independently of the type and values’ distribution of the feature involved.

Among the possibilities of feature selection algorithms, an modification of the well-known Markov Blanket feature selection (MBFS) [10] algorithm adapted for continuous features [11,12] was used.

The MBFS method is an iterative methodology that returns a relevance ranking of the input features with respect to the classification result, taking to account, not only their importance, but also the redundancy among themselves. The main difference of this algorithm with respect to other well-known MI based algorithms, such as mRMR [36] or NMIFS [37], is that it performs a backward feature selection. MBFS eliminates the least important feature in each iteration of the algorithm, instead of adding the most relevant feature in each step. This has the advantage of not eliminating features that themselves might not provide information, but together with other features do, leading to an improvement in truth relevance identification.

## 4. Results

This section presents the results that were obtained for the classification of the type of primary from the available dataset.

All of the features were normalized to have zero mean and unit standard deviation. As it was aforementioned dataset was first randomly shuffled and subdivided in 80% of the data for training and validation purposes for hyperparameter optimiztion (under a five cross-validation scheme), and the remaining 20% for test. The the whole dataset was repeatedly validated in a different five cross-validation scheme for performance assessment (see Section 3.1.5), providing mean and std over training and test performances.

All of the methods were implemented under Python, using Keras, XGBoost, and Sklearn libraries, and executed under a Intel corei7 32GBRAM PC with NVIDIA GeForce GTX 10800 GPU.

### 4.1. Classification

Two main feature settings were evaluated according to the demands of the Theoretic Physics experts. Later, results using the NMIFS ranking were assessed in order to provide this information to the experts. The two sets were:

1. 5 features: NALLParticles, MUTotal, ELTotal, Zenith, Energy
2. 3 features: MUTotal, Zenith, Energy

**Figure 1:**
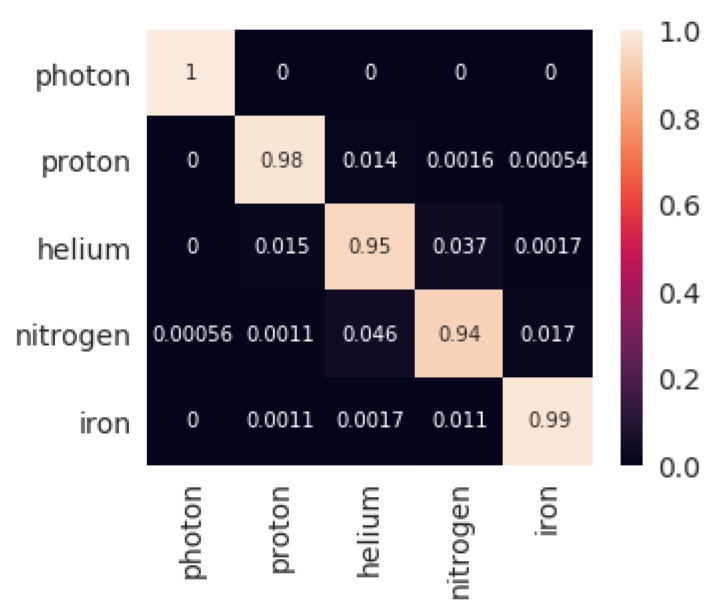
Confusion matrix for the first test set returned by XGBoost classification with five features.

**Figure 2:**
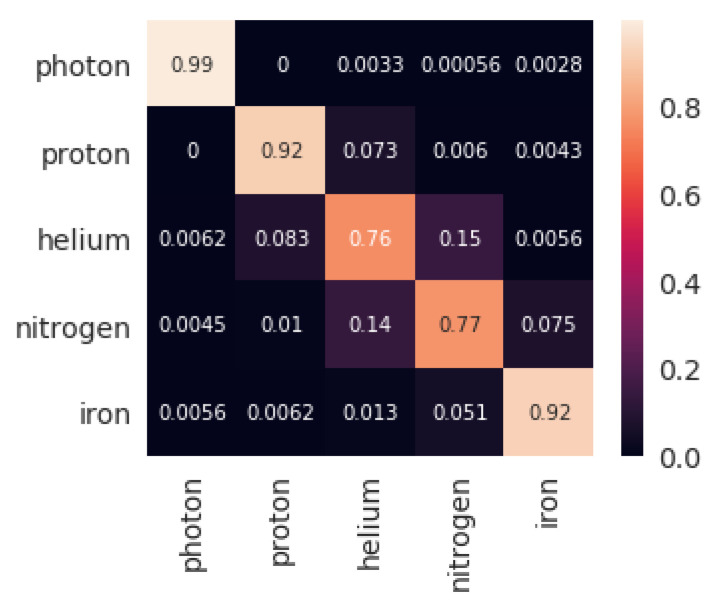
Confusion matrix for the first test set returned by XGBoost classification with three features.

The results obtained by the four classifiers are shown in Table 1. Both sets with five and three features were assessed. The training times for each of the classification methods included the training of the hyper-parameters of the model. Hyperparameters for each of the classification models using a first training-test subdivision of the dataset are shown in Table 2. The confusion matrix obtained by XGBoost for both settings for that initial subdivision (highest accuracy, as seen in Table 1) are shown in Figure 1 and Figure 2.

### 4.2. Feature Ranking

**Figure 3:**
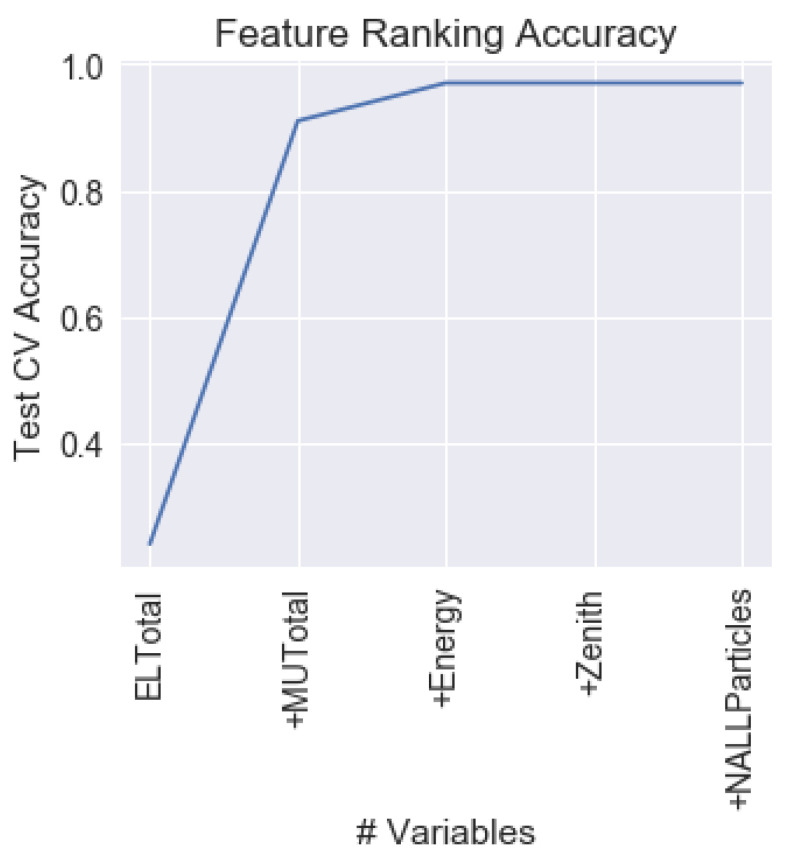
Evolution of the test performance on the problem according to the ranking returned by the Markov Blanket Mutual Information Feature Selection (MBFS) algorithm using XGBoost. Hyperparameters of XGBoost were optimized for each feature subset size combination.

The MBFS algorithm using Kraskov Mutual Information estimation algorithm was executed on the training set, returning the following feature ranking (from most relevant to lowest relevant feature): ELTotal, MUTotal, Energy, NALLParticles, and Zenith. Figure 3 shows the evolution of the test performance as from one to five features are considered, using XGBoost algorithm. As it can be seen, with only two features, the ELTotal + MUTotal results surpass the 0.9 of test performance. Nevertheless, with only one feature, accuracy is surprisingly low (0.24). It is important to also highlight that the MI of the ELTotal feature with respect to the classification feature showed to be very similar to the MI between the MUTotal feature and the classification feature. Moreover, tests performed using MUTotal as single feature for classification showed comparable results (0.24) to those using ELTotal as a single feature to perform the classification. Moreover, Figure 3 shows that, with the first three features, performance is similar than using all of them. This implies that information of Zenith angle and NAllParticles becomes irrelevant after considering ELTotal, MUTotal, and Energy.

It is important to highlight that algorithms mRMR and NMIFS failed to recognize the optimal ranking retourned by MBFS algorithm. NMIFS attained the identification of the two most relevant features (ELTotal + MUTotal), failing to identify the third one (Energy, identified in fifth position and, thus, only attaining the 97% of accuracy using five features). mRMR, on the other hand, missidentified the essential relationship between ELTotal and MUTotal, identifying as second most relevant feature the Zenith angle, leading to 0.9 of accuracy only after using three features.

## 5. Discussion

After showing the results that were obtained in the comparison, this Section discusses it. This comparison is made when considering both the classification accuracy and the computing cost.

Regardless of the type of particle, XGBoost presents an outstanding performance, being very precise in classifying all of them (as Figure 1 and Figure 2 show). This methodology seems superior to the other alternatives not only because it obtains the best results but because the model is trained in shorter time (just behind KNN). The next suggested methodology is SVM, being very close to XGBoost in classification metrics, but with executions 20 times slower. DNNs are next in the classification accuracy, but it is the slowest technique by far (100 times slower than XGBoost). Finally, the KNN algorithm presents disappointing classification scores, but it is clearly the fastest methodology (100 times faster than XGBoost). The performance ranking obtained by the four methodologies for five features is similar to that obtained for three features. For the latter, XGBoost and SVM achieved a comparatively better result than the other two methodologies, undergoing a lower performance decrease when taking away two of the input features.

Comparing these results with the preliminary work in [7], in which DNNs were applied, then the best accuracy for five variables reached 0.94, and 0.82 for three variables, while using an optimzed DNN model after several tests, and a single training-test subdivision. Thus, the results were similar, taking into account mean and standard deviation seen in Table 1. The optimal model then attained the best results while using four layers, while the CV scheme in this work leads to a reduction in the complexity of the network by selecting a two-layered network. In any case, as seen, SVM and XGBoost attained a faster training and better performance than DNNs for the problem tackled.

For the sake of fairness, the comparative was designed, so that, for all the models, the number of combinations of hyperparameters was similar, as explained in Section 3. Table 2 shows the optimal hyperparamenter configurations for the four techniques, for the two feature subsets considered. SVMs and KNN obtained the same values for the hyperparameters for both feature subsets. XGBoost maintained the maximum depth, but the eta parameter presented a larger value for five features. This seems expected, as the weights of the features can shrink more when their number is smaller. Analogously, DNN preserves the two layered configuration (we should keep in mind that, even with one layer, they could be universal approximators), but with a lower number of units per layer for three input features. For the second case, the architecture of the network becomes much simpler.

The reason why the DNNs consumed much more time than XGBoost is because the training was carried out when considering up to 80 different architectures with a maximum of 500 epochs. This might be one of the main flaws of these models in comparison with the other approaches, the cost of finding the right set of hyperparameters might be too expensive. When considering that SVMs are restricted by the number of samples, from the computational cost perspective, the best choice is XGBoost.

In relation to KNN, it is important to highlight that this work utilized its simplest version in the optimization. KNN may sometimes suffer from the presence of noisy features or by differences in the relevance of the features involved. However, despite that features were equally normalized, its performance was lower than the other methods. Although KNN model optimization was simpler than for the other methods (only k was optimized for a single distance metric), and could include feature weights optimization, for instance, in this work a specific feature selection process was performed as a separated next step, whose results are shown in Section 4.2.

Once the best technique has been observed, it is possible to take a closer look to the results of XGBoost. Figure 1 and Figure 2 show the confusion matrices that were obtained by the algorithm when using 5 and 3 features respectively. When using all the features: NALLParticles, MUTotal, ELTotal, Zenith, Energy, the capability of separating Photons from the rest of particles is perfect. The accuracy remains outstanding, even in the subgroup of Hadrons. Although, as the matrix shows, very light and very heavy particles, such as Proton and Iron, respectively, are classified perfectly, but the particles in between (Helium and Nitrogen in this work) some misclassifications are shown. This last observation becomes even more dramatic when the number of features is reduced to: MUTotal, Zenith, and Energy. Photons, Protons, and Irons are quite well classified, but there is an important source of errors coming from Helium and Nitrogen classification, which fall to a 76% of correct labelling.

The results obtained by the MBFS ranking are surprising, because, by using only two features, the results obtained are better than by using the three features suggested by the experts. This is interesting, because it motivates the research considering the electromagnetic part of the signal instead of considering uniquely the muonic component. Additionally, by using the three most relevant features MUTotal, ELTotal, and Energy, the results attained are similar than using the five of them, implying the irrelevancy of NALLParticles and Zenith after considering the first three. Analyzing the data coming from the simulator, it is observed that almost all of the information about the cascade development is contained in the electromagnetic (ELTotal) and muonic (MUTotal) components of the shower. With the additional information on the primary energy (Energy), it is possible to obtain the three most relevant information of the event: energy, mass composition, and direction of arrival. Therefore, the results obtained by MBFS corroborate the results that were obtained by the simulator of cascade.

## 6. Conclusions

There are many unknowns in Astrophysics and, to be able to determine the composition of the UHECR, might help to understand some mechanisms that rule the Universe. This paper has presented a comparative analysis of several machine learning techniques in order to see how precise it possible to carry out the classification of the particle composition of a cosmic ray. To the sight of the results, XGBoost has shown an outstanding performance, also being efficient from the computational load point of view. The results obtained by performing a data driven approach suggest that considering the information of both the muonic and electromagnetic component instead of just one of them improves the solution to the problem under study. This consideration might be even more important when the trace of the event is available.

## Figures and Tables

**Table 1:** Classification report obtained by the classification approach with five and three features over test dataset.

	5 Features			3 Features		
	trn. Time (s.)	Accuracy	f1-Score	trn. Time (s.)	Accuracy	f1-Score
ANN	48,715	0.91 (0.015)	0.92 (0.012)	23,957	0.76 (0.14)	0.77 (0.017)
XGBoost	909	0.97 (0.002)	0.97 (0.002)	843	0.87 (0.002)	0.87 (0.002)
SVMs	9536	0.94 (0.003)	0.94 (0.003)	10,677	0.83 (0.004)	0.83 (0.004)
KNN	3.59	0.78 (0.003)	0.79 (0.003)	2.75	0.62 (0.006)	0.63(0.005)

**Table 2:** Hyperparameters obtained for each classification approach with five and three features.

Classifier	5 Features	3 Features
ANN	2 layers, n.u.=[39,31]	2 layers, n.u.=[17,18]
XGBoost	maxdepth=5,eta=0.85	maxdepth=5,eta=0.55
SVMs	σ=512,γ=0.5	σ=512,γ=0.5
KNN	k=1	k=1
